# Supplementary material for: Overcoming Microenvironment-Mediated Chemoprotection through Stromal Galectin-3 Inhibition in Acute Lymphoblastic Leukemia
Source: Int J Mol Sci. 2021 Nov 10;22(22):12167. doi: 10.3390/ijms222212167 (PMC8624256; doi:10.3390/ijms222212167)
Supplement: Supplementary file 1 [file ijms-22-12167-s001.zip › ijms-1418521-supplementary.pdf]

Tarighat et al. Overcoming microenvironment-mediated chemoprotection through stromal galectin-3 inhibition in acute lymphoblastic leukemia

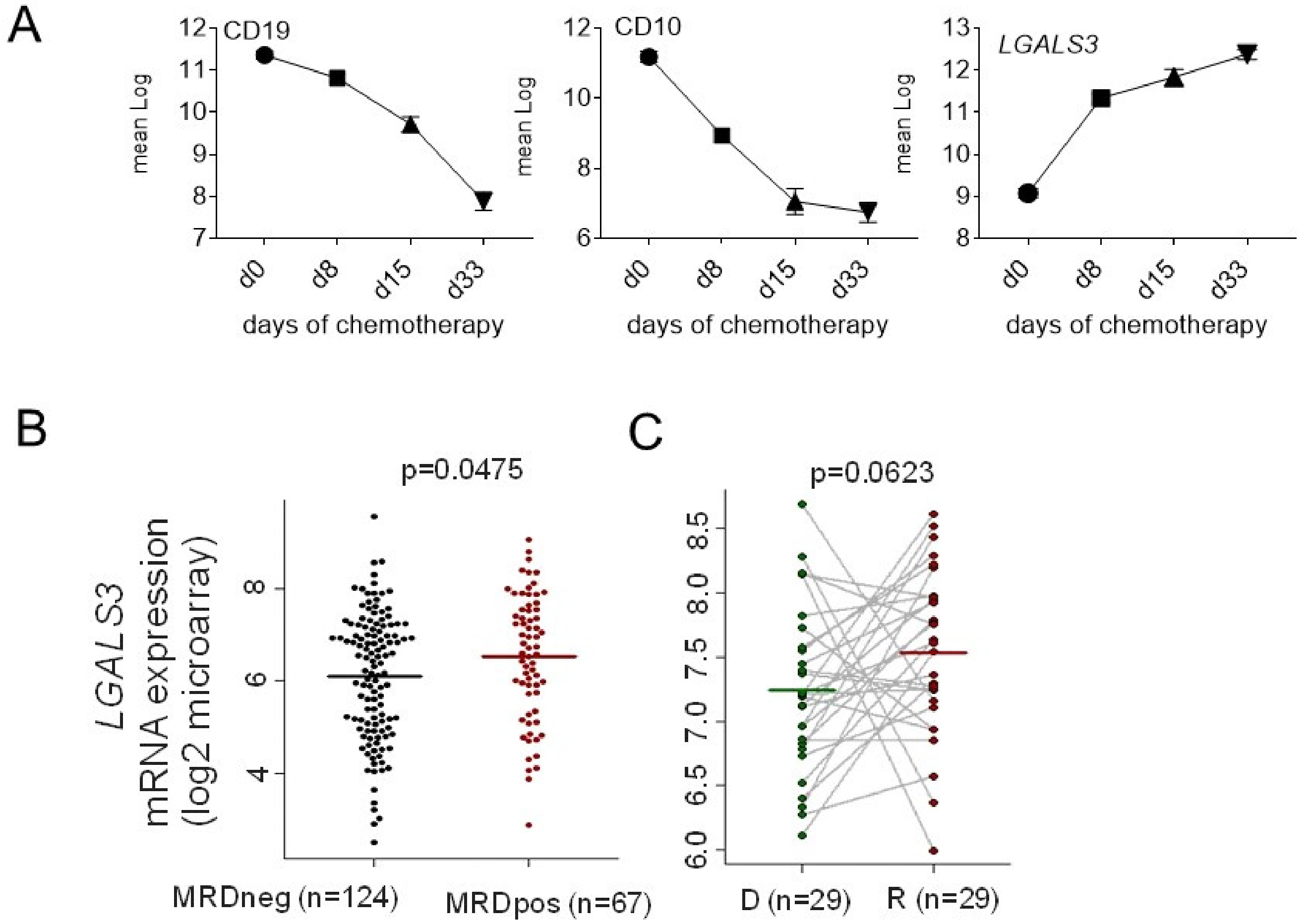

Figure S1. High levels of *LGALS3* mRNA in pediatric BCP-ALL. (**A**) Mean log-transformed normalized GEP values for the indicated genes on 220 pediatric de novo ALL at diagnosis, day 8, day 15, and day 33 of remission-induction therapy (23) (GSE67684). (**B**) *LGALS3* expression for probe set 1557197_a_at in the indicated samples [gene microarrays, log2 expression, COG P9906, GSE11877). p-value, two-sided Wilcoxon test. (**C**). *LGALS3* in paired early (<36 months) relapse and diagnosis pediatric ALL samples (Gene microarray array expression (log2) COG P9906 GSE28460). p-values, paired two-sided Wilcoxon test.

Tarighat et al. Overcoming microenvironment-mediated chemoprotection through stromal galectin-3 inhibition in acute lymphoblastic leukemia

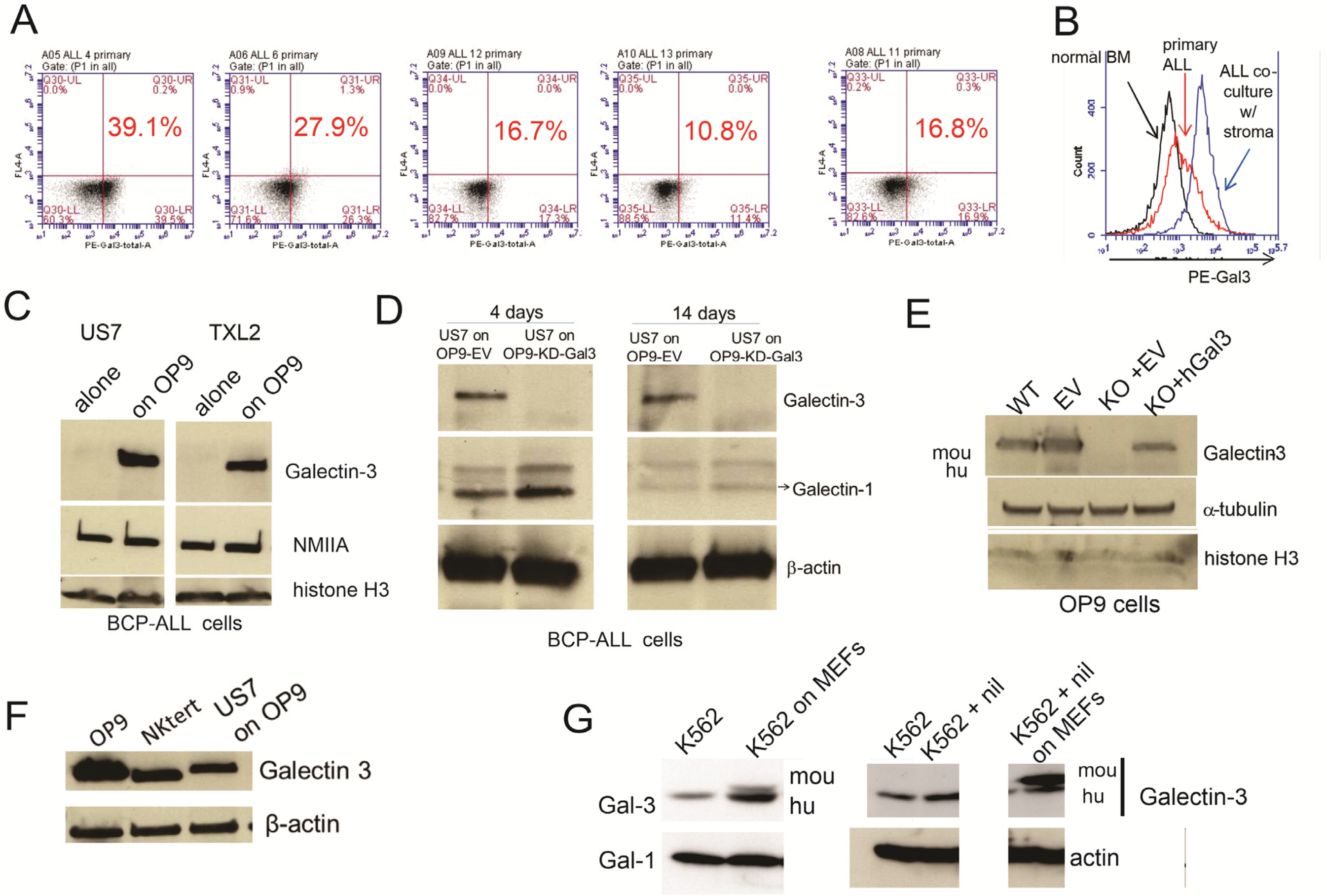

H

```
CLUSTAL O(1.2.4) multiple sequence alignment
SP|P17931|LEG3_HUMAN MADNFSLHDALSGSGNPNPQGWPGAWGNQPAGAGGYPGASYPGAYPGQAPPGAYPGQAPP 60
SP|P16110|LEG3_MOUSE MADSFSLNDALAGSGNPNPQGYPGAWGNQP-GAGGYPGAAYPGAYPGQAPPGAYPGQAPP 59
                     ***.***:***:*********:******** ********:********************

SP|P17931|LEG3_HUMAN GAYPGA--------------------PGAYPGAPAPGVYPGPPSGPGAYPSSGQPSATGA 100
SP|P16110|LEG3_MOUSE GAYPGQAPPSAYPGPTAPGAYPGPTAPGAYPGQPAPGAFPGQPGAPGAYP-----QCSGG 114
                     *****                     ****** ****.:** *..*****     ..:*.

SP|P17931|LEG3_HUMAN YPATGPYGAPAGPLIVPYNLPLPGGVVPRMLITILGTVKPNANRIALDFQRGNDVAFHFN 160
SP|P16110|LEG3_MOUSE YPAAGPYGVPAGPLTVPYDLPLPGGVMPRMLITIMGTVKPNANRIVLDFRRGNDVAFHFN 174
                     ***:****.***** ***:*******:*******:**********.***:**********

SP|P17931|LEG3_HUMAN PRFNENNRRVIVCNTKLDNNWGREERQSVFPFESGKPFKIQVLVEPDHFKVAVNDAHLLQ 220  ← hu
SP|P16110|LEG3_MOUSE PRFNENNRRVIVCNTKQDNNWGKEERQSAFPFESGKPFKIQVLVEADHFKVAVNDAHLLQ 234  ← mou
                     **************** *****:*****.**************** **************
```

Figure S2. Endogenous galectin-3 expressed in BCP-ALLs in comparison to stromal-produced galectin-3. (**A**) Total Gal3 expression in 5 different primary BM BCP-ALL samples using PE-Gal3 antibodies. Ficoll-purified mononuclear cells viably frozen in LN_2 were thawed, washed and kept for 3 hrs in α-MEM before fixation / permeabilization. Red numbers, % of Gal3-positive cells based on gating set by control IgG. (**B**) FACS histogram of total Gal3 expression in primary leukemia sample #11 (Ph-, >90% CD10/CD19+) on day 0 and after ~10 days co-culture with irradiated OP9 stroma cells, compared to normal bone marrow on day 0. (**C**) Western blot of human US7 or TXL2 BCP-ALL cells that had been kept in medium without OP9 stromal cells ('alone') or co-cultured with OP9 wild type stromal cells. (**D**) Western blot of US7 cells co-cultured with OP9 –

EV or OP9- Gal3 knockout cells for 4 or 14 days as shown. (**E**) Western blot of four different OP9 stromal variants as shown. KO+ EV are OP9 cells with galectin-3 knockout and also transduced with empty vector pMIG; KO+hGal3 are OP9 cells with galectin-3 knockout and transduced with human HA-tagged galectin-3 expressing vector pMIG. Mou, hu: murine galectin-3 migration on SDS-PAA gels is slightly slower compared to human galectin-3. (**F**) Western blot of mouse OP9 stromal cells, human NKtert stromal cells and US7 cells that had been in co-culture with OP9 stromal cells. Note the more rapid migration of human galectin-3 in the human NKtert cells and the location of the galectin-3 in the US7 cells consistent with its murine origin. (**G**) Western blot analysis of K562 cells grown alone or with irradiated MEFs overnight as indicated. Antibodies are indicated to the left and right of the panels. Actin, or galectin-1: loading control. Nilotinib, 1 µM nilotinib for 24 hrs. Note that K562 expresses galectin-3 endogenously. Human galectin-3 migrates below mouse galectin-3 on SDS-PAA gels consistent with the difference in primary sequence shown in panel H sequence alignment of human galectin-3 and murine galectin-3, indicating the human protein is 14 amino acids shorter. Panels E and F adapted from https://doi.org/10.1101/2021.09.22.461145

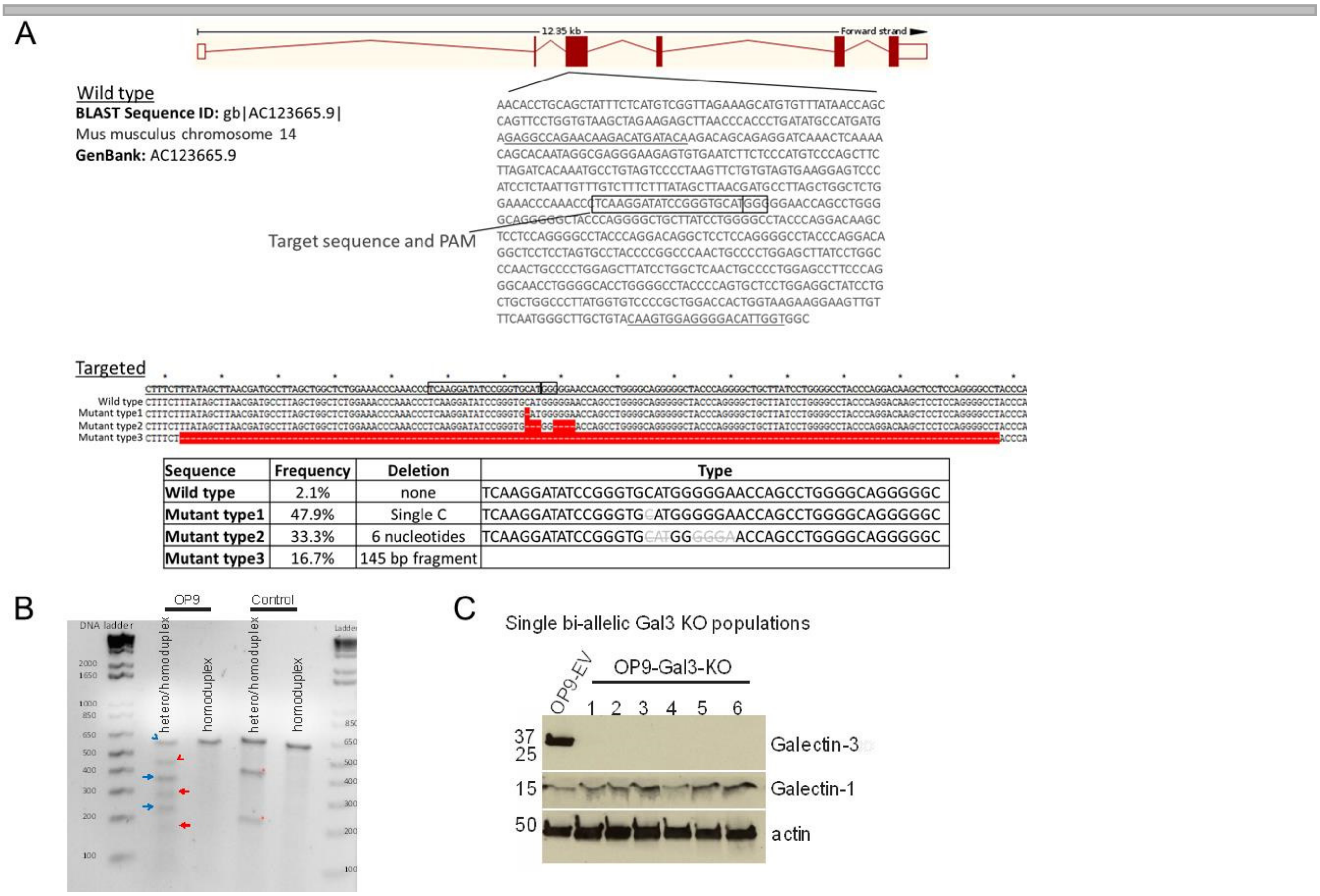

Sequence	Frequency	Deletion	Type
Wild type	2.1%	none	TCAAGGATATCCGGGTGCATGGGGAACCAGCCTGGGGCAGGGGGC
Mutant type1	47.9%	Single C	TCAAGGATATCCGGGTGCATGGGGAACCAGCCTGGGGCAGGGGGC
Mutant type2	33.3%	6 nucleotides	TCAAGGATATCCGGGTGCATGGGGGAACCAGCCTGGGGCAGGGGGC
Mutant type3	16.7%	145 bp fragment	

Figure S3. CRISPR/Cas9 mediated genome editing to knock out Gal3 in OP9 cells. (**A**) The CRISPR/Cas9 target region in exon 3 of the *lgals3* gene. The OP9-Gal3-KO has nucleotide deletion compared to the wild-type gene; this leads to a frameshift missense mutation and premature stop codons downstream in exon 3. (**B**) Surveyor assay to detect DNA breaks induced by CRISPR/Cas9 in exon 3 of OP9 cells. (**C**) Western blot showing Gal3 knockout in OP9 clones carrying bi-allelic CRISPR-induced mutation.

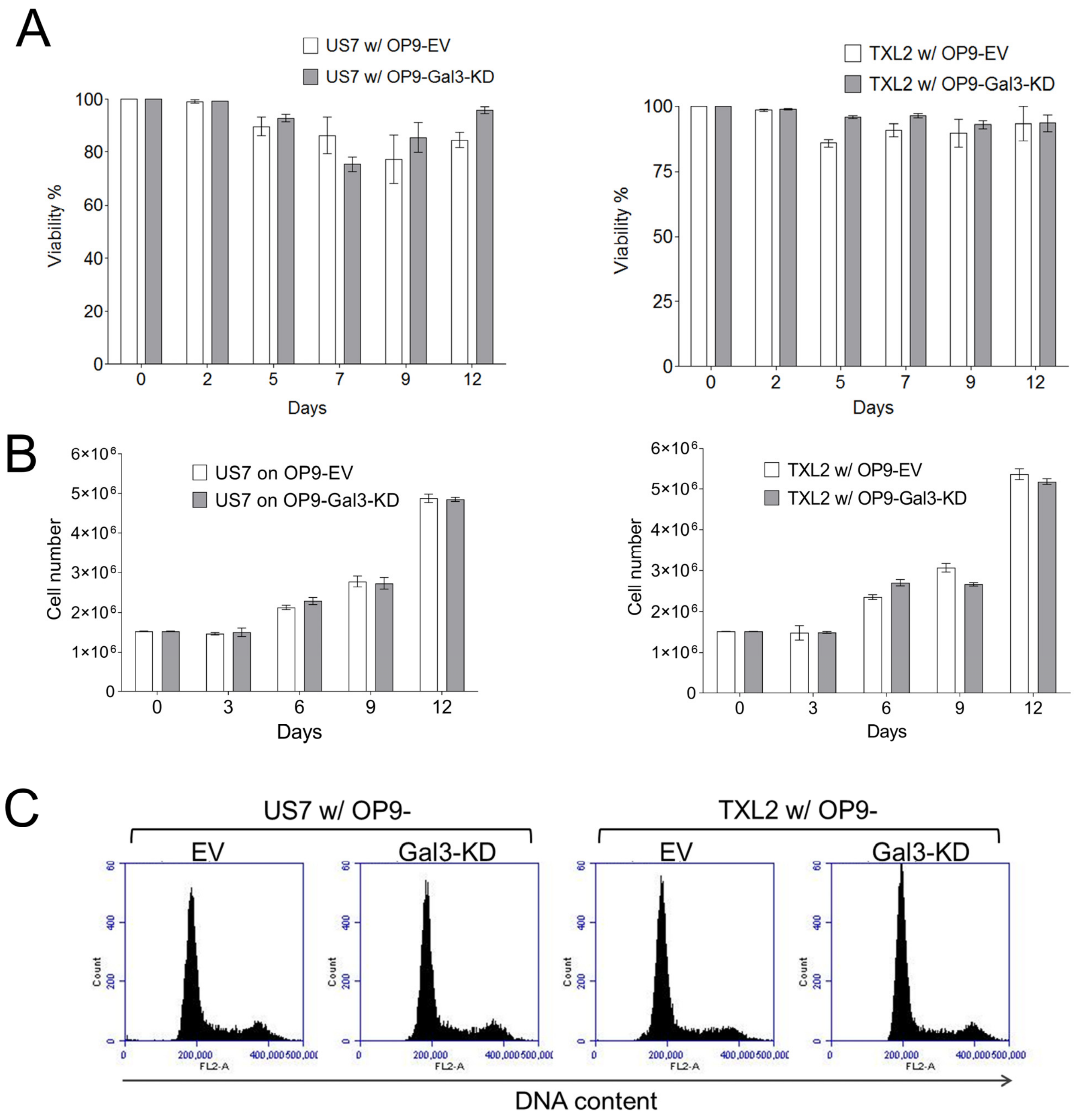

Figure S4. Under steady-state growth, galectin-3 produced by stromal cells is not essential. (**A**) Viability and (**B**) cell counts of US7 [left] and TXL2 [right] cells measured by Trypan blue exclusion grown over 12 days on the indicated OP9 stromal cells. (**C**) Cell cycle analyzed by FACS and DNA content at different phases of the cell cycle in US7 or TXL2 cells grown for more than 4 days in co-culture with the indicated OP9 cells.

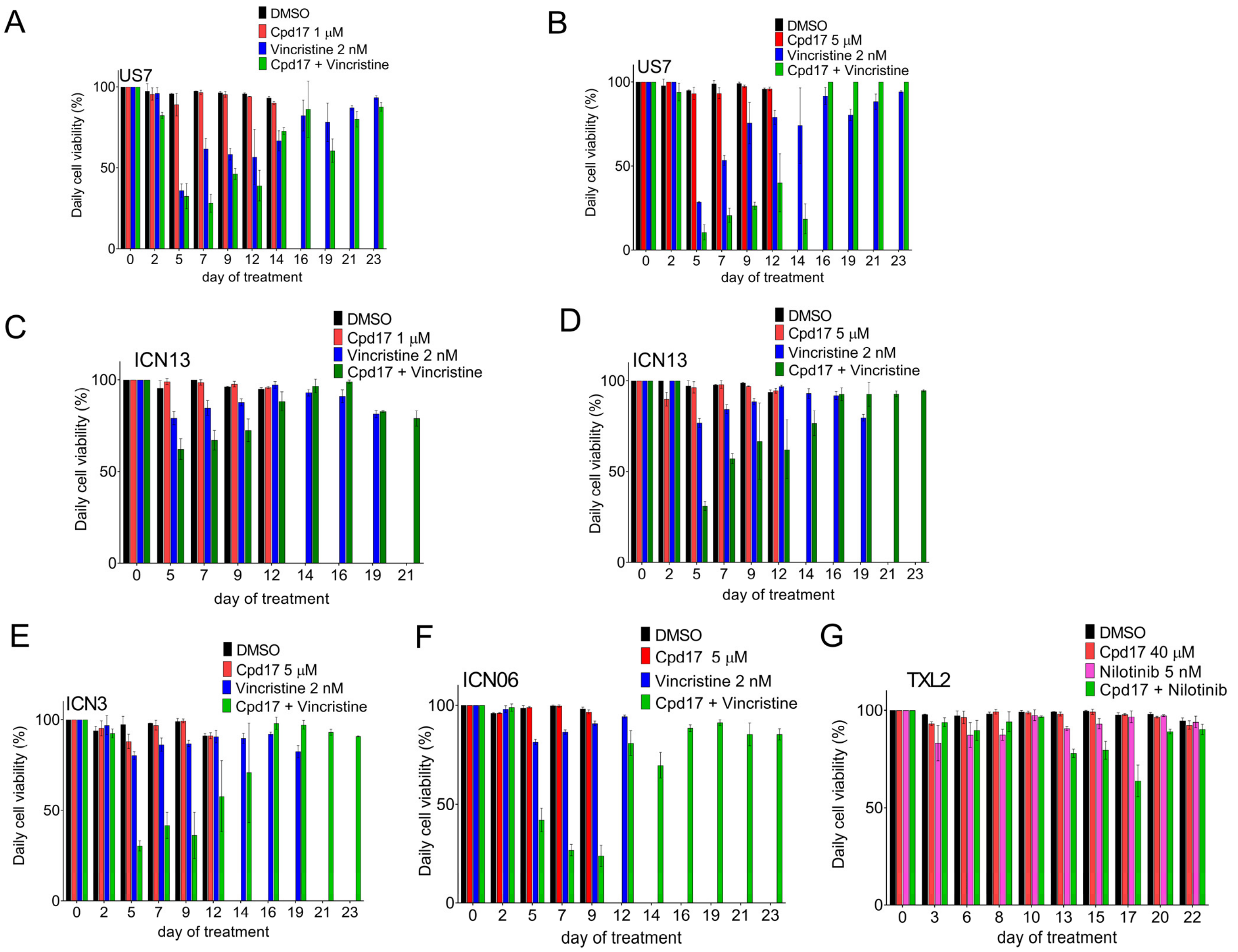

Figure S5. Viability of BCP-ALLs treated with combination treatment including Cpd17. Samples (Fig. 8) were treated with solvent DMSO [black bars], Cpd17 [red bars], standard chemotherapy [blue bars] or a combination of chemotherapy with Cpd17, [green bars]. DMSO only, Cpd17 mono-treatment and vincristine mono-treatment samples were not followed after a certain period when cell numbers exceeded the capacity of the wells and cultures became overgrown in A-F. Viability = viable cell count/total cell count x 100%. This is a measurement for the Trypan-blue excluding [living] status of cells that remain in the culture, even if they are only very few in number. Error bars, mean ±SEM of 2-4 wells. Fresh drugs at the same concentration were added with every medium change. However, in panel G, Cpd17 was administered at 10 µM on day 0, increased to 20 µM on day 6 and further increased to 40 µM on day 10 because drug combination effects were minimal at 10 and 20 µM.

A-F: 2 nM vincristine as standard chemotherapy;
G: 5 nM targeted tyrosine kinase inhibitor nilotinib.
A-B: US7 cells; C-D: ICN13; E: ICN3; F: ICN06; G: TXL2.
A, C: 1 µM Cpd17.
B, D-F: 5 µM Cpd17.
G: 40 µM Cpd17 from d10 onwards.
